# miR-19a-3p promotes the growth of hepatocellular carcinoma by regulating p53/SOX4

**DOI:** 10.1016/j.heliyon.2024.e36282

**Published:** 2024-08-13

**Authors:** Hang Zhang, Jiajun Zhu, Jingjun Zhang, Ying Liu, Baicheng Zhao, Xiaoyi Yang, Wenhan Zhou, Bozhou Chen, Shuangshuang Zhang, Ruotong Huang, Shuying Chen

**Affiliations:** a Department of Laboratory Medicine, Huashan Hospital, Fudan University, 12 Wulumuqi Middle Road, Shanghai, 200040, China; b Medical College, Fudan University, 130 Dongan Road, Shanghai, 200032, China; c Department of Rehabilitation Medicine, The Sixth People's Hospital Affiliated to Shanghai Jiao Tong University School of Medicine, Shanghai, 200233, China

**Keywords:** Hepatocellular carcinoma, miR-19a-3p, p53, SOX4, Mechanism, Clinical target

## Abstract

**Objective:**

This study aims to investigate the potential functions of miR-19a-3p in HCC.

**Method:**

We collected serum samples to analyze miR-19a-3p expression. We utilized CCK8 and Transwell assays to access miR-19a-3p′s influence on HCC cells malignancy. We used dual-luciferase reporter and western blotting to validate the impact of p53/miR-19 on miR-19/SOX4.

**Results:**

The results demonstrated that miR-19a-3p was highly expressed in pre-operative serum samples and HCC cells, which can promote cell proliferation, migration and invasion in HCC under *in vitro* conditions. Additionally, there was a p53 binding site on the upstream of miR-19a-3p, which was inhibited by p53. SOX4 was the direct gene targeted by miR-19a-3p. The imbalance of p53-miR-19-SOX4 loop was one reason for the progress of HCC.

**Conclusion:**

Our findings validate the mechanisms of miR-19a-3p and highlight its potential as a therapeutic target in HCC.

## 1 Introduction

Hepatocellular carcinoma (HCC) is one of the most common malignant tumors of the digestive tract in humans [1,2]. Almost half of new hepatocellular carcinoma cases globally come from China each year. The high metastasis and invasiveness rate of HCC contribute to poor prognosis of HCC patients. The five-year recurrence rate after surgery is 50 %–70 % [3]. Therefore, it has become one of the major topics of discussion in the tumor research domain to clarify the mechanisms of the occurrence and progress of hepatocellular carcinoma and find effective biological markers for preliminary screening, diagnosis and prognostic monitoring of HCC [4,5].

MicroRNAs (miRNAs) were non-coding endogenous small RNAs with a length of close to 22 nucleotides, playing a crucial role in gene regulation [6]. miRNAs modulate gene by specifically interacting with the 3′UTR of mRNAs. The level of base pairing between the miRNA and its target mRNA determines the effectiveness of gene repression. Exact matches usually lead to mRNA degradation, while partial matches induce translational repression. In addition, both the near-seed and non-seed regions of the miRNA are necessary for its ability to regulate gene expression. These mechanisms enable miRNAs to affect a diverse variety of cellular functions, involving cell differentiation, proliferation, and apoptosis. Recently, researches have shown that miRNAs contribute to cancer progression. Nuclear miRNAs can affect gene expression by mediating gene silencing or activation [7]. In many cancer types, miRNA expression profiles have changed significantly, and some miRNAs are considered to be tumor suppressors or oncogenes. These miRNAs affect tumor growth, infiltration, and metastasis via the regulation of cancer-related signaling pathways [8,9]. Many miRNAs are reported to be abnormally expressed in hepatocellular carcinoma. Among these, miR-23c, miR-139, miR-185, miR-199b, and miR-612 have anti-tumor effects [[10], [11], [12], [13], [14]], while miR-106b, miR-665 and miR-5692a play roles in promoting cancer [[15], [16], [17]]. Additionally, the changes of miR-21, miR-299, miR-638 and miR-7706 families can serve as prognostic indicators for liver cancer [[18], [19], [20]]. Therefore, studying the mechanisms and functions of miRNAs in cancer holds great importance in clarifying the nature of cancer and developing new curative strategies.

miR-19a-3p, as a key member of the miR-17-92 cluster, has been discovered to be associated with various malignancies such as breast cancer, pancreatic cancer, lung cancer, cervical cancer, and gastrointestinal malignancies [[21], [22], [23], [24], [25]]. It has been indicated that miR-19a-3p functions as a tumorigenic gene and is markedly upregulated in liver cancer in comparison with nearby healthy tissues. Furthermore, miR-19a-3p may stimulate liver cancer metastasis and chemoresistance through targeting PTEN [26]. Nevertheless, the functions of miR-19a-3p in HCC remain to be elucidated. p53 is among the most extensively studied oncosuppressor proteins related to almost all cancers, including hepatocellular carcinoma [27]. Studies have demonstrated that p53 can suppress the proliferation and tumorigenicity of HCC through mediating cell cycle arrest [28]. The chemoresistance of hepatocellular carcinoma can be mediated by the mutation of p53, and p53 can also be used as a biological marker for new HCC after direct-acting antivirals (DAA) treatment [29,30]. Phosphorylated p53 sering-15 is also a biomarker for the survival and prognosis of patients with hepatocellular carcinoma [31]. Nevertheless, the impact of p53 on miR-19a-3p in HCC is not very explicit, and how miR-19a-3p and p53 interact further require to be elucidated.

The SOX protein family, as a new-discovered transcription factor family, contributes importantly to the progression of cancers [[32], [33], [34]]. SOX4 is a member of the C subfamily within the SOX family and also one of the most important regulators of p53, which predominantly affects the function of p53 in three ways. SOX4 can activate the response of p53 to DNA damage through ATM/ATR/DDR. SOX4 can also regulate the stability of p53 protein and increase its transcriptional activity. In addition, some investigations have revealed that SOX4 can increase the protein level of p53 by blocking p53 ubiquitination mediated by MDM2. Feedback regulation is an important means of maintaining homeostasis in organisms, which can prevent overregulation in cells [35]. In normal cells, the feedback regulation between p53 and SOX family is one of the most important regulation modes.

In our study, we discovered that miR-19a-3p is markedly elevated in the serum of HCC patients. Mechanistically, we find that miR-19a-3p inhibits the functions of tumor cell. We also found that p53 could directly or indirectly inhibit the expression and maturation of miR-19a-3p transcriptionally. Moreover, it can, in turn, regulate the activity of p53 through its impact on the expression of the SOX4 family, thus forming a feedback regulation loop and amplifying the anti-tumor activity of p53. Considering that, we believe that the feedback regulation loop of p53-miR-19-SOX4-p53 has an extremely significant role in the normal physiological functions of cells. In HCC, the feedback regulation loop is broken, and the cell enters a vicious circle, which promote the occurrence and progress of HCC. Therefore, revealing the specific molecular mechanism of p53/miR-19/SOX4 in HCC and restoring the function of p53→miR-19→SOX4→p53 feedback regulation cycle may help the adjuvant therapy of HCC.

## 2 Materials and methods

### 2.1 Samples

Both preoperative and postoperative serums were gathered from 30 patients clinically diagnosed as HCC at Huashan Hospital (Shanghai, China) from July 2022 to September 2022. Additionally, we also collected serum specimens of 30 healthy individuals as controls. These controls had no known malignant tumors or active inflammatory conditions and were matched with gender, age and race of HCC patients. The pre-collected serum specimens were centrifuged at a speed of 4000 rpm, and the supernatant was preserved at −80 °C. Clinical characteristics of the 30 healthy examinees and 30 HCC patients are presented in Table 1. There were no discernible variations in age, gender, alanine aminotransferase (ALT), and aspartate aminotransferase (AST) distribution between the HCC patients and healthy individuals, except for viral infection and AFP.

**Table 1:** Clinicopathological parameters of subjects.

Parameters		Health	HCC	*P* value	χ^2^
Total		30	30		
Gender	Male Female	13 17	14 16	0.795	0.067
Age (years old)	<60 ≥60	14 16	14 16	1.000	0.000
ALT (U/L)					
	<40	18	13	0.196	1.669
	≥40	12	17		
AST (U/L)					
	<34	15	13	0.605	0.268
	≥34	15	17		
Viral infection					
	Positive	2	24	0.000***	32.851
	Negative	28	6		
AFP (ng/ml)					
	0–20	28	3	0.000***	41.018
	＞20	2	27		

*** *P* < 0.001.

### 2.2 Cell culture

Normal human liver epithelial cells (L02) and hepatoma cell lines (HepG2, Huh7, SMMC-7721 and MHCC97H) were obtained from American Type Culture Collection (ATCC). The genetic testing biotechnology company used short tandem repeat (STR) analysis to perform cell line authentication on the cell lines involved in the experiments. Mycoplasma contamination was determined through MycoAlert Mycoplasma detection to confirm the absence of mycoplasma. 10 % fetal bovine serum (FBS) added to DMEM was used to cultivate cells.

### 2.3 Cell transfection

MiR-19a-3p inhibitors and miR-NC were purchased from RiboBio (Shanghai, China). 50 nM RNA oligonucleotides were diluted in 250 μL Opti-MEM medium along with the appropriate amount of Lipofectamine 2000. After gentle mixing, the solution was incubated for 20 min at room temperature to form a complex. This complex was then incubated with 1 × 10^5^ cells for 6 h. Subsequently, the cells were placed into fresh medium and harvested at specified time points following transfection. Stable expression was achieved using lentivirus-mediated transfection.

### 2.4 Proliferation assay

A 96-well plate was utilized to seed stably transfected cells (1 × 10^5^ cells per well). On days 1, 2, 3, and 4, each well received 10 μL CCK-8 solution (Dojindo, Japan), followed by a 2-h incubation period and overnight culture. Subsequently, the absorbance at 450 nm was recorded to determine cell growth.

### 2.5 Migration assay

Transwell was used to assess cell migration following the manufacturer's guidelines. 1 × 10^5^ cells was placed into the upper of a Boyden Chamber, which contains an 8 μm polycarbonate membrane. Below the membrane, 600 μL FBS was added. And then the chambers were maintained in the incubator for 24 h. Migrated cells were treated with 4 % paraformaldehyde for half an hour. Then they were treated with 0.1 % crystal violet solution for 10 min. Photomicrographs were captured with a microscope (Olympus, Japan), and the migrated cells were counted from six randomly chosen sections.

### 2.6 Invasion assay

Transwell chamber was precoated with Matrigel Matrix (BD Biosciences, USA). On upper chamber, 1 × 10^5^ cells was added, while 600 μL FBS was supplemented to the bottom chamber. After 48 h, the upper chamber cells were removed. For quantitative analysis, the cells passing through the membrane were processed using 4 % paraformaldehyde, treated with 0.1 % crystal violet stain, and photographed.

### 2.7 Dual-luciferase reporter assay

Online databases (TargetScan, miRBase and Pic Tar) were employed to predict genes targeted by miR-19a-3p. RT-PCR was used to amplify DNA fragment corresponding to SOX4 mRNA 3′-UTR and then the fragment was inserted into the p-MIR-reporter vector (as a control group). In contrast, a site-directed mutagenesis PCR method was used to construct the mutant 3 ʹ -UTR of the SOX4 with miR-19a-3p target site. For luciferase reporter tests, HEK293 cells were cultured in 96-well plates (Promega Corporation), and then Lipofectamine 2000 with a luciferase reporter vector was used to conduct a transient co-transfection with miR-19a-3p mimic or control microRNA (5′-UUCUCCGAACGUGUCACGUTT-3′). A dual-luciferase reporter was carried out to detect the transcription function of construct promoter after transfection at 48 h.

### 2.8 Quantitative RT-PCR (RT-qPCR)

RNA was extracted from serum by the miRNeasy Serum/Plasma kit (Qiagen, Germany) referring to the manufacturer's manual. Total RNA was obtained from the cells by the MiRNeasy Mini kit (Qiagen, Germany). The sequence of miR-19a-3p is 5′-UGUGCAAAUCUAUGCAAAACUGA-3’. MiR-19a-3p expression in cells was analyzed by the MiScript SYBR-Green PCR kit (Qiagen, Germany). Expression of cel-miR-39 (5′-UCACCGGGUGUAAAUCAGCUUG-3′) and RNU6B (5′-GCTTCGGCAGCACATATACTAAAAT-3′) functioned as endogenous controls in the serum and cell tests respectively. Cel-miR-39 originates from the nematode *Caenorhabditis elegans* and does not share homology with human miRNA. Therefore, it can serve as an exogenous control to detect and correct variability in experimental procedures. The expression of cel-miR-39 in serum samples is typically stable and not influenced by many external factors, such as blood handling processes or storage conditions. RNU6B is a common RNA molecule in human cells closely related to cellular biology status. Thus, it serves as a naturally occurring endogenous control. The expression of RNU6B in cells is relatively stable, making it a reliable internal control for comparing miRNA expression under different conditions. The classical 2^- ΔΔCt^ approach was performed to evaluate and normalize the data. Primers for miR-19a-3p, cel-miR-39 and RNU6B were provided by Qiagen.

### 2.9 Western blotting

Cells were lysed on ice in IP lysis buffer with phosphatase inhibitors and protease (Beyotime, China) for half an hour, followed by centrifuging cell lysates (12,000 g, 4 °C, 30 min). The protein samples were taken, and concentration of protein was measured by BCA Kit (Beyotime, China). Lysates buffer was necessary to dilute the protein sample to 5 μg/μl for protein data normalization. Protein (10 μg) was separated and transferred to the PVDF membrane (Millipore) by 12 % SDS-PAGE. Following blocking with skim milk, blots were incubated with anti-β-actin protein and Rabbit anti-Human SOX4 below 4 °C. The incubation was done overnight. The Goat anti-Rabbit secondary antibody was utilized to wash an incubate membranes for 60 min, followed by a BeyoECL Plus kit (Beyotime) aiming at demonstrating color.

### 2.10 Animal procedures

Male BALB/c nude mice aged 4–5 weeks (Shanghai Laboratory Animal Center, China), were arranged under special pathogen-free (SPF) conditions for one week for acclimatization. 5 × 10^6^ HepG2/p53 WT and HepG2/p53 MUT cells were administered to the nude mice from both sides of the posterior flanks to construct p53 wild-type and mutant mice. At 0 h, 24 h, and 48 h, all mice were exposed to radiation (IR; 8 Gy) to induce p53 expression. The relative expression of miR-19a-3p was also measured.

HepG2 is a human hepatocellular carcinoma cell line with highly differentiated liver cell characteristics. These cells are easy to culture and have been widely used in many laboratory studies as a model for liver cancer. Since they are isolated from liver cancer patients, HepG2 cells retain many characteristics of liver cancer, making them a valuable tool for studying the pathophysiology, biology, and treatment methods of liver cancer. Additionally, BALB/c nude mice are a strain of mice lacking an immune system, thus they cannot produce a natural anti-tumor response. This makes them an ideal model for evaluating xenograft tumor transplantation. In liver cancer research using nude mice, HepG2 cells can be transplanted into these mice either subcutaneously or into the liver, thereby creating a liver cancer model. This model allows researchers to evaluate multiple biological activities like liver cancer cell growth, metastasis, and response to treatment. Overall, using HepG2 cells and BALB/c nude mice to construct an animal model of liver cancer provides a reliable and reproducible platform for studying the pathogenesis, treatment methods, and effectiveness of new therapeutic strategies for liver cancer.

### 2.11 Statistical analysis

The SPSS 19.0 (SPSS Inc, USA) was applied to perform data analysis. T test, one-way analysis of variance (ANOVA) or Mann-Whitney's test were adopted to process the quantitative data. The data correction was performed using the Bonferroni method for correction. A Chi-square test was performed for the qualitative data. Data was demonstrated as mean ± SD. The experiment was conducted a minimum of three times. Statistical significance was considered if bilateral p values were less than 0.05.

## 3 Results

### 3.1 Upregulated miR-19a-3p in serums and cells of hepatocellular carcinoma

**Fig. 1:**
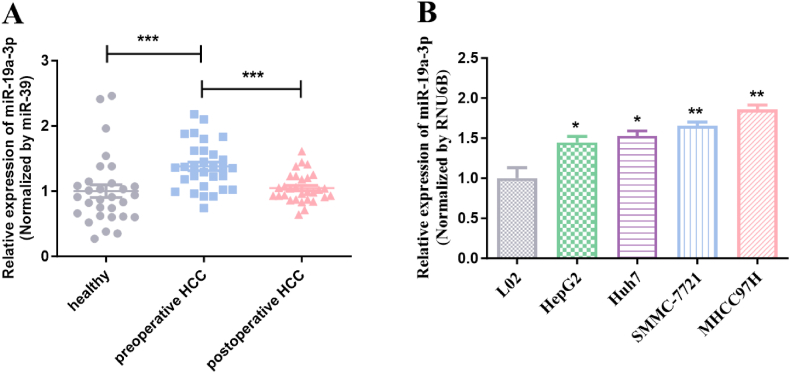
Relative expression level of miR-19a-3p in HCC serum and cells. (A) MiR-39 acts as an internal control. qRT-PCR is used to investigate the expression level of miR-19a-3p in serum samples. (B) Fluorescent quantitative real-time PCR is applied to investigate the expression level of miR-19a-3p in normal liver epithelial cells (L02) and hepatocellular carcinoma cell lines (HepG2, Huh7, SMMC435 7721, MHCC97H). *P < 0.05, **P < 0.01, ***P < 0.001.

To ascertain if there is a statistically significant difference in miR-19a-3p between healthy and HCC specimens, serums from 30 healthy individuals and 30 HCC patients both before and after surgery were gathered. RNA was extracted from each group's serum. The results indicate that miR-19a-3p expression in the pre-operative serum of the 30 HCC patients was statistically higher than that in the serum of healthy and post-operative patients (Fig. 1A). RNA was also extracted from cells. The results, shown in the figure, indicate that miR-19a-3p expression was comparatively elevated in hepatocarcinoma cell lines (Fig. 1B).

### 3.2 The biological functions of HCC cells can be affected by miR-19a-3p *in vitro*

**Fig. 2:**
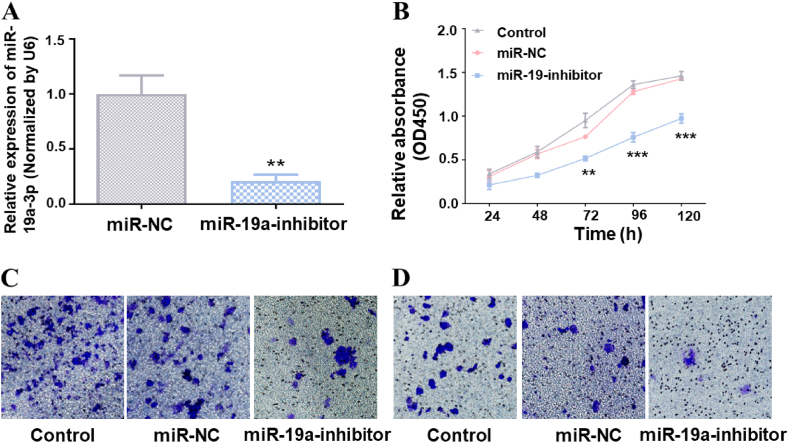
Suppression of the miR-19a-3p expression level can hinder the growth, migration and invasion of HCC. (A) A hepatocellular carcinoma cell line stably expressing miR-19 inhibitor was constructed. The expression level of miR-19a-3p expression in the HepG2 stable cell line was investigated using qRT-PCR, with RNU6 serving as an internal control. (B) The impact of miR-19a-3p on the proliferation of HepG2 cells are assesses using a microplate reader to measure proliferation activity at 450 nm every 24 h. (C) The effect of miR-19a-3p on the migration function of HepG2 cells was analyzed using a Transwell assay. After 24 h, the upper chamber was stained by 0.1 % crystal violet, and observations were made using a microscope. (D) The effect of miR-19a-3p on the invasion of HepG2 cells was evaluated using the Matrigel method. After 48 h, the upper chamber was stained by 0.1 % crystal violet, and observations were made using a microscope. **P < 0.01, ***P < 0.001.

To verify the roles of miR-19a-3p in the proliferation, migration and invasion of HCC cells. We constructed hepatocellular carcinoma cell lines stably expressing miR-19a inhibitor (Fig. 2A). As shown in the figure, a statistical significant reduction in cell proliferation was detected in HepG2 stably expressing miR-19 inhibitor (Fig. 2B). Additionally, the transwell assay and Matrigel method indicated that the expression of the miR-19 inhibitor hindered the migration and invasion of HCC cells (Fig. 2C and D). Generally, miR-19a-3p can be considered a tumor-promoting factor in hepatocellular carcinoma.

### 3.3 The expression of miR-19a-3p is inhibited by p53

**Fig. 3:**
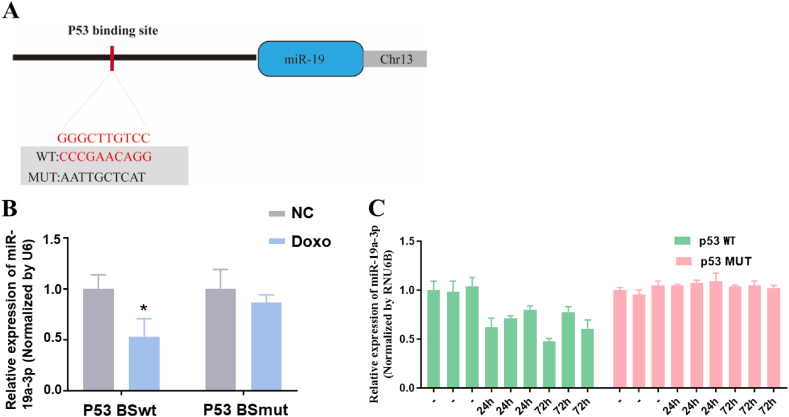
p53 inhibits the expression of miR-19a-3p at the transcriptional level. (A) A schematic diagram shows a latent p53 binding site 2.2 kb upstream of miR-19 transcription start site. (B) After transfecting the miR-19 promoter reporter plasmid containing the p53 binding site or mutation site into HepG2 cells, the relative fluorescence intensity was measured. (C) Both p53 wild-type mice and p53 mutant mice were exposed to radiation (IR; 8 Gy) to induce the expression of p53. After that, the relative expression of miR-19 was measured. *P < 0.05.

To verify whether p53 can downregulate miR-19a-3p expression, we defined the miR-19a-3p promoter using UCSC Genome Browser. We then predicted the p53 binding location, which was 2.2 kb upstream of the miR-19a-3p transcription start site (Fig. 3A). To further confirm that p53 can bind to the upstream site of miR-19a-3p transcription, a miR-19a-3p promoter reporter plasmid containing p53 binding sites and mutation sites was constructed. HepG2 cells were then transfected with this plasmid. We found that the p53 agonist (doxorubicin) inhibited luciferase activity. However, the p53 agonist with mutations in the p53 binding site had no effect, indicating that p53 can bind to the predicted binding site (Fig. 3B). Additionally, we irradiated p53 wild-type mice and p53 mutant mice with radiation (IR; 8Gy) to induce p53 expression. We then detected the miR-19a-3p relative level normalized by RUN6B. The results showed a notable decline in miR-19a-3p levels in p53 wild-type mice, while miR-19a-3p expression in p53 mutant mice did not change significantly (Fig. 3C). In conclusion, these results strongly suggest that p53 inhibits miR-19a-3p levels in HCC cells.

### 3.4 MiR-19a-3p directly targets SOX4 in hepatoma cell

**Fig. 4:**
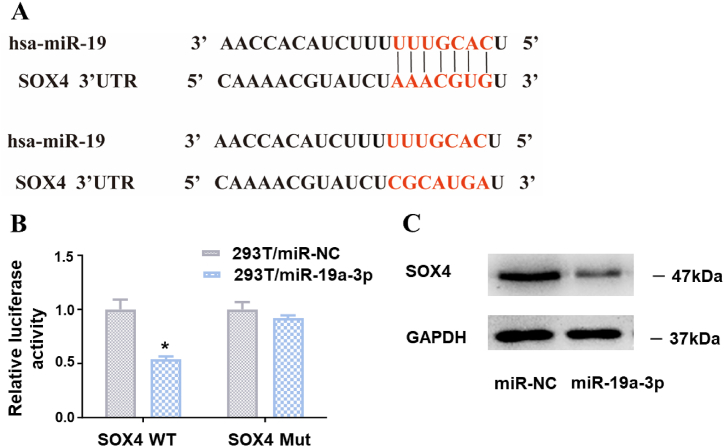
The direct transcriptional regulation effect of miR-19a-3p on SOX4 expression. (A) The putative miR-19a-3p binding site is located in the 3ʹ-UTR of SOX4. (B) RT-PCR was used to amplify the 3′-UTR fragment of SOX4, which was then cloned into the PMIR-LUC report gene vector. A mutant reporter gene plasmid was constructed by introducing non-matching nucleic acid into the predicted binding site of miR-19a-3p within the SOX4 3′-UTR. Both the wild type (WT) and mutant (Mut) reported gene plasmids were confirmed by sequencing. Luciferase reporter gene assays were used to evaluate the relative luciferase activity of HEK-293T cells co-transfected with miR-19a-3p or a scrambled control (SCR). (C) Immunoblotting assays were used to detect the protein levels of SOX4 (see Figs. S1 and S2 in supplementary Content for the full, non-adjusted image).*P < 0.05.

The interaction between SOX4 and p53 upregulated p53 expression and enhanced its function. First, we screened miR-19a-3p targets using bioinformatics tool such as Targetscan and Pictar. We then validated whether SOX4 was a direct protein targeted by miR-19a-3p (Fig. 4A). Next, HEK-293T cells were simultaneously transfected with miR-19/scrambled control (SCR) and a reporter gene plasmid, followed by a reporter gene assay. It turned out that overexpressing miR-19a-3p inhibited the function of the wild-type SOX4 reporter gene, while the activity of the mutant plasmid remained unaffected. This suggests that the 3′-UTR fragment of SOX4 can be bounded by miR-19 (Fig. 4B). The binding site matched our predicted binding sequence. Further immunoblotting assays verified that overexpressing miR-19 in HCC cells significantly downregulated SOX4 expression at the protein level (Fig. 4C). The full, non-adjusted image is provided in Supplementary Figs. S1 and S2. Thus, we substantiated that miR-19 may regulate SOX4 by binding to its 3′- UTR fragment directly.

## 4 Discussion

Research has verified that miRNAs take part in the biological activity of hepatocellular carcinoma [36,37]. The abnormal expression of miRNA can drive the development of HCC through the regulation of cancer stem cells (CSCs) self-renewal, accelerating the cell cycle to enhance the proliferation of HCC cells, increasing the chemical resistance of hepatoma cells, stimulating the migration of liver cancer cells, and promoting tissue colonization to advance the progress of HCC [38]. HCC is caused by the imbalance of various signal pathways, including mitochondrial-mediated apoptosis, cell division and proliferation controlled by cell cycle checkpoints, and signaling pathways activated by receptor tyrosine kinases (RTKs), among others. It has been confirmed that miRNAs serve a critical function in the intricate network associated with the progression of HCC [39]. Moreover, the detection of certain miRNA levels can help in predicting HCC metastasis and recurrence [40]. MiRNAs might develop into innovative biomarkers and may offer innovative evidence for distinguishing between hepatocellular carcinoma and liver metastatic tumor [41]. As one of the miRNA molecules, miR-19a-3p has been identified to participate in different biological processes in numerous cancer tissues [42,43]. However, there is still limited research on the related mechanism of miR-19a-3p in HCC. Comprehending the molecular processes involving miR-19a-3p in the occurrence and progression of hepatocellular carcinoma remains urgent.

In this work, we collected pre-operative and post-operative serum samples from 30 healthy subjects and 30 patients with HCC. We determined that miR-19a-3p exhibited significant overexpression in the pre-operative serum of HCC patients. Additionally, we observed that miR-19a-3p can promote the malignant behavior of hepatocellular carcinoma *in vitro*, including migration, invasion and proliferation. Since the tumor suppressor p53 regulates a variety of non-coding RNAs through complex pathways [44,45], we explored the potential of p53 in regulating miR-19a-3p and confirmed that p53 could directly regulate miR-19a-3p expression transcriptionally using a luciferase reporter gene assay. This helps us better understand the relationship of p53 and miR-19a-3p in the occurrence and progression of hepatocellular carcinoma.

As an important tumor suppressor gene, p53 is regulated by many factors, including SOX4 [46]. We first predicted that miR-19a-3p can target SOX4 and then identified SOX4 as the direct gene targeted by miR-19a-3p using luciferase assay. We discovered that overexpressed miR-19a-3p can reduce the protein level of SOX4. Moreover, we observed that miR-19a-3p expression was elevated in hepatocellular carcinoma serum and cells. Suppressing the expression of miR-19a-3p can restrain the progression of hepatocellular carcinoma. A interaction site of p53 upstream of miR-19a-3p was identified, allowing p53 to suppress miR-19a-3p expression. Additionally, SOX4 is directly regulated by miR-19, and miR-19 can suppress SOX4 expression. All these findings strongly suggest that there is a p53-miR-19-SOX4 feedback loop in HCC cells, and the disruption of this circuit is engaged in the progression of HCC.

However, the techniques and methods used in this study may have some potential limitations and variations. The efficiency of transfection reagents could be a key factor affecting gene transfection and expression. We have tried multiple transfection reagents and optimized transfection conditions to achieve the best transfection efficiency. However, we also acknowledge that even under optimal conditions, there may still be some variability in transfection efficiency, which could impact the experimental results. Additionally, we have conducted detailed exploration and experimentation in the selection and optimization of cell culture conditions to ensure the health and stable growth of cells. However, we recognize that different cell types and culture conditions may have varying effects on gene expression. Therefore, we have fully considered these potential variations in our research and made corresponding adjustments in experimental design and analysis. There are also some limitations in our study. Studies have proved that several non-coding RNAs with dysregulated expression were closely involved in the chemical resistance of HCC which has not been covered in our study [47]. Moreover, p53 protein can play a crucial part in tumor tissues through a variety of signal pathways [48,49], but we did not explore the specific molecular biological interaction mechanism of p53, SOX4 and miR-19 in this study. In addition, in clinical practice, the high genetic heterogeneity of liver cancer may be the main cause of treatment failure [50]. The traditional tumor cell lines were used in this study, which may not fully represent clinical related diseases because of the genetic heterogeneity. In the future, through patient-derived xenograft models or different cell lines, this weak point would be corrected. Next, we also plan to conduct further experiments in animal models to validate the role of the miR-19, p53, and SOX4 feedback loop in disease progression, along with the possible therapeutic benefits of regulating this pathway. Based on our preliminary findings, we will further explore the feasibility of targeting miR-19 or its targets as therapeutic strategies. We plan to design and test modulators of miR-19a-3p or other related drugs to evaluate their impact on disease treatment outcomes. We will also consider designing clinical trials to assess the safety, efficacy, and feasibility of potential treatment strategies. Through clinical research, we hope to gain deeper insights into the potential value of this novel pathway in the treatment of human diseases.

## 5 Conclusion

This study revealed that miR-19a-3p expression was up-regulated in HCC serum and cells, and inhibiting miR-19a-3p expression can restrain the progression of HCC. Comprehensive study of the molecular pathway discovered a binding site for p53 upstream of miR-19a-3p and p53 lowered miR-19a-3p expression at the transcription level. Moreover, SOX4 was confirmed as a direct target of miR-19a-3p. The expression of SOX4 can be suppressed by miR-19a-3p. Our study supported the potential application value of miR-19a-3p in HCC. It can not only enhance the proliferation, migration and invasion of hepatocellular carcinoma cells but also participate in the feedback loop between p53 and SOX4. This indicates that there is a relationship between the p53-miR-19-SOX4 feedback loop and the oncogenic behavior of hepatocellular carcinoma cells, and miR-19 may have the potential to be used as a biological marker of HCC in clinical settings. This study provides new insights and approaches for the development of microRNA-targeted therapy technology and serves as a supplement to the understanding of the mechanisms relevant to the onset and progression of hepatocellular carcinoma.

## Ethics statement

This study was approved by the Human Research Ethics Committee of Huashan Hospital affiliated to Fudan University (KY2022-533). Written informed consent was obtained from all the participants. All animal experiments were approved by the National Institutes of Health Guide for the Care and Use of Laboratory Animals and were approved by the Animal Experimental Ethics Committee of Fudan University (2021JS-121).

## Data availability statement

Data will be made available on request. If you would like to obtain the data, please contact the corresponding author.

## CRediT authorship contribution statement

**Hang Zhang:** Writing – original draft, Methodology, Investigation. **Jiajun Zhu:** Writing – original draft. **Jingjun Zhang:** Writing – review & editing, Writing – original draft. **Ying Liu:** Writing – review & editing, Resources, Formal analysis. **Baicheng Zhao:** Formal analysis, Data curation. **Xiaoyi Yang:** Methodology, Formal analysis. **Wenhan Zhou:** Writing – original draft, Funding acquisition. **Bozhou Chen:** Writing – original draft, Formal analysis. **Shuangshuang Zhang:** Writing – review & editing, Writing – original draft. **Ruotong Huang:** Validation, Methodology, Investigation. **Shuying Chen:** Writing – review & editing, Conceptualization.

## Declaration of competing interest

The authors declare that they have no known competing financial interests or personal relationships that could have appeared to influence the work reported in this paper.
