# Supplementary material for: miR-19a-3p promotes the growth of hepatocellular carcinoma by regulating p53/SOX4
Source: Heliyon. 2024 Aug 13;10(16):e36282. doi: 10.1016/j.heliyon.2024.e36282 (PMC11381758; doi:10.1016/j.heliyon.2024.e36282)
Supplement: Multimedia component 1 [file mmc1.docx]

**miR-19a-3p promotes the growth of hepatocellular carcinoma by regulating p53/SOX4**

Hang Zhang^a,b,1^, Jiajun Zhu^a,b,1^, Jingjun Zhang^c,1^, Ying Liu^b^, Baicheng Zhao^b^, Xiaoyi Yang^a^, Wenhan Zhou^b^, Bozhou Chen^a^, Shuangshuang Zhang^a^, Ruotong Huang^a,b^, Shuying Chen^a*^

^a^*Department of Laboratory Medicine, Huashan Hospital, Fudan University, 12 Wulumuqi Middle Road, Shanghai 200040, China*

^b^*Medical College, Fudan University, 130 Dongan Road, Shanghai 200032, China*

^c^*Department of Rehabilitation Medicine, The Sixth People's Hospital Affiliated to Shanghai Jiao Tong University School of Medicine, Shanghai 200233, China*

^*^ Corresponding author. Department of Laboratory Medicine, Huashan Hospital, Fudan University, 12 Wulumuqi Middle Road, Shanghai 200040, China.

*E-mail addresses:* shuyingchen@fudan.edu.cn (S. Chen).

^1^ HZ, JZ and JZ contributed equally as co-first authors.

**Supplementary Figures**





Figure S1. The full, non-adjusted image of GAPDH detected by western blotting.





Figure S2. The full, non-adjusted image of SOX4 detected by western blotting.
